# Violent victimization among immigrants: Using the National Violent Death Reporting System to examine foreign-born homicide victimization in the United States

**DOI:** 10.1016/j.pmedr.2022.101714

**Published:** 2022-01-29

**Authors:** Kayla R. Freemon, Melissa A. Gutierrez, Jessica Huff, Hyunjung Cheon, David Choate, Taylor Cox, Charles M. Katz

**Affiliations:** a Center for Violence Prevention and Community Safety, Arizona State University, Phoenix, AZ, USA; b School of Criminology and Criminal Justice, University of Nebraska Omaha, Omaha, NE, USA; c Department of Criminal Justice, University of Texas at El Paso, El Paso, TX, USA

**Keywords:** Foreign-born, Homicide, Immigration, NVDRS

## Abstract

Limited research attention has focused on homicides involving foreign-born victims. Using data from the National Violent Death Reporting System, we examined 9428 homicides that occurred in 2017 in the United States across 32 states and D.C. Approximately 8% of homicide victims were foreign-born. Homicide victimization rates were substantially lower for foreign-born persons, compared to U.S.-born persons. However, foreign-born persons from Honduras, El Salvador, and Jamaica had a substantially higher risk of homicide victimization. Notably, few homicides involving foreign-born victims were gang- or drug-trade-related. With the growing number of immigrants in the United States, policy and prevention efforts should be guided by research.

## 1 Introduction

The foreign-born population comprises about 14% of all residents in the United States (Radford, 2019), which is near the all-time high reached in the early 1900 s (Dews, 2018). In fact, in 1990, about 20 million foreign-born persons were residing in the U.S., compared to about 45 million in 2017 (Migration Policy Institute, n.d.). A recent Gallop poll reported that 23% of Americans believe that immigration is the second most important problem facing the nation behind only “the government” at 26% (Jones, 2019).

The foreign-born population largely comprises two groups: lawful immigrants (77%) and unauthorized immigrants (23%; Budiman, 2020). Despite the increasing foreign-born population in the U.S., relatively little is known about the prevalence of violent victimization among foreign-born individuals. This is a notable concern given that prior research has found that immigrants, both lawful and unauthorized, who experience violence fear seeking medical attention (Grace et al., 2018) and are less likely to report victimization to the police because of their immigration status (Cepeda et al., 2012). As such, understanding the true extent of violent victimization among immigrants, in addition to risk and protective factors, is challenging.

Given limitations in using official healthcare and police data to assess victimization among foreign-born populations, several studies have relied on self-reported victimization. For instance, a study of undocumented immigrants in Los Angeles and Philadelphia indicated that fear of deportation was an important factor when considering whether to contact the police in response to crime (Armenta and Rosales, 2019). Considering these findings, it is clear that violent victimization among the foreign-born population poses a concern, though using traditional data sources might undercount these incidents.

Homicide, however, is often considered one of the best measures of victimization because these incidents nearly always come to the attention of authorities. Research from the 1970s through the 1990s reported that foreign-born persons were at higher risk of homicide victimization than those born in the U.S. (Sorenson and Shen, 1996, Singh and Hiatt, 2006); however, more recent research suggests that this gap has narrowed (Singh and Hiatt, 2006). Research has been largely limited to general trends in homicide and victimization, with little focus on the underlying circumstances contributing to these incidents. Understanding these factors will inform whether foreign-born individuals might be disproportionately victimized during some circumstance-specific killings, identifying important avenues to prevent these incidents.

Little research has provided theoretical insight into victimization differences between U.S.-born and foreign-born populations. Prior work has focused on how acculturation experiences influence victimization with immigrants becoming more accultured over time as well as how such experiences increase their offending, particularly through weakened family bonds and cultural values, in turn impacting victimization (Sommers et al., 1994). Alternatively, some have suggested that immigrants are less likely to be victimized because their lifestyles and routine activities decrease risk factors (Eggers and Mitchell, 2016).

The present study examines the scope and nature of foreign-born homicide victimization in the U.S. using data from the 2017 National Violent Death Reporting System (NVDRS). The NVDRS is a violent death surveillance system sponsored by the Centers for Disease Control and Prevention (CDC). We explore the prevalence of foreign-born homicide victimization overall and by nation of birth. We also examine the characteristics of foreign-born homicide victims and incident characteristics through multilevel modeling at the state-level. These results have important implications for understanding victimization among individuals who could be undercounted in violence data and for identifying risk factors among this population.

## 2 Methods

### 2.1 Data

The present study uses data contained in the 2017 NVDRS Restricted Access Database (RAD)^1^. The NVDRS links data from death certificates, coroners/medical examiners, and law enforcement agencies to provide a comprehensive view of fatal violent incidents. As of 2017, 35 states, the District of Columbia (D.C.), and Puerto Rico participated in the NVDRS^2^. The NVDRS defines homicide as “a death resulting from the intentional use of force or power, threatened or actual, against another person, group, or community” (CDC, 2018, p. 9). We only examine homicides, excluding legal interventions, suicides, and other types of violent deaths.

The 2017 NVDRS RAD includes a total of 13,669 homicides. We excluded cases from Puerto Rico, Illinois, Pennsylvania, and Washington as those states only collected data on a portion of violent deaths, and we were unable to link their sample with the state total population to calculate rates (n = 2,736). California restricts data collection to violent deaths occurring in four counties (Los Angeles, Sacramento, Shasta, and Siskiyou); any violent deaths reported outside of those counties were excluded for the same reason as noted above (n = 1,229)^3^. We also removed cases when the geographic location of the homicide was unknown or missing (n = 104). We reviewed case narratives when the victim’s birthplace was unknown or missing (n = 164); birthplaces were identified in seven cases, and the remaining cases were excluded. Finally, we removed cases outside of the sample states (n = 15). A total of 9,428 cases remained for analysis from 32 states and D.C. The NVDRS data were supplemented with 2017 5-year estimates from the U.S. Census Bureau's American Community Survey (ACS) for the purpose of calculating homicide rates^4^. IRB approval was granted by Arizona State University.

### 2.2 Measures

#### Dependent variable

Victims born in the U.S. or in a territory where the U.S. grants citizenship at birth, including Guam, Puerto Rico, Northern Mariana, and the U.S. Virgin Islands (U.S. Census Bureau, 2019), were coded as U.S.-born (=0). Victims not born in the U.S. or U.S. territories were coded as foreign-born (=1). Descriptive statistics are displayed in Table 1.

**Table 1:** Descriptive statistics (N = 9,428).

		n (%)
Foreign-born	Yes	730 (7.74)
	No	8,698 (92.26)
Age	0–14	459 (4.87)
	15–24	2,288 (24.27)
	25–34	2,692 (28.55)
	35–54	2,757 (29.24)
	55 and up	1,232 (13.07)
Sex	Male	7,431 (78.82)
	Female	1,997 (21.18)
Marital status	Married	1,521 (16.13)
	Not married	7,792 (82.65)
	Unknown	115 (1.22)
Race/Ethnicity	White	2,804 (29.74)
	Black	4,809 (51.01)
	Asian/Pacific Islander	156 (1.65)
	Other/unspecified	298 (3.16)
	Hispanic	1,358 (14.40)
	Unknown	3 (0.03)
Education	Below high school	3,107 (32.96)
	High school degree/GED	4,178 (44.31)
	Some college credit	1,061 (11.25)
	Associate degree	398 (4.22)
	Bachelor degree/higher	511 (5.42)
	Unknown	173 (1.83)
Mental health diagnosis	Yes	444 (4.71)
	No	8,984 (95.29)
Alcohol problem	Yes	333 (3.53)
	No	9,095 (96.47)
Substance abuse problem	Yes	1,117 (11.85)
	No	8,311 (88.15)
Victim–suspect relationship	Current or former partner	888 (9.42)
	Family member	780 (8.27)
	Acquaintance	1,950 (20.68)
	Stranger	597 (6.33)
	Other	37 (0.39)
	Unknown	5,176 (54.90)
Method/weapon	Firearm	6,763 (71.73)
	Sharp instrument	1,042 (11.05)
	Blunt object	451 (4.78)
	Hanging, strangulation	249 (2.64)
	Other	714 (7.57)
	Unknown	209 (2.22)
Location of injury	House, apartment	4,412 (46.80)
	Street, sidewalk, alley	1,941 (20.59)
	Public use area	752 (7.98)
	Other	1,837 (19.48)
	Unknown	486 (5.15)
Victim used a weapon	Yes	503 (5.34)
	No	8,925 (94.66)
Gang-related	Yes	733 (7.77)
	No	8,695 (92.23)
Homicide precipitated by another crime	Yes	2,326 (24.67)
	No	7,102 (75.33)
Nature of the othercrime (most serious)	Drug trade	283 (12.17)
	Robbery	612 (26.31)
	Burglary	260 (11.18)
	Motor vehicle theft	57 (2.45)
	Arson	24 (1.03)
	Rape, sexual assault	51 (2.19)
	Gambling	7 (0.30)
	Assault, homicide	844 (36.29)
	Witness tampering	11 (0.47)
	Other	137 (5.89)
	Unknown	40 (1.72)

Other Race/Ethnicity includes American Indian/Alaska Native (n = 157), Two or more races (n = 112), and Other/unspecified non-Hispanic (n = 29).

#### Independent variables

**Victim characteristics.** We discuss victim demographic characteristics at an individual level, including a categorical measure of age, sex (male = 1, female = 2), marital status (married = 1, not married = 0), race/ethnicity, education, and binary measures of diagnosis of any mental health problem and of alcohol or other substance abuse problems (yes = 1, no = 0)^5^.

**Incident and circumstance characteristics.** Incident characteristics included a categorical measure of the relationship between the victim and the offender, whether the victim used a weapon (yes = 1, no = 0), the categorical method/weapon used to perpetrate the homicide, the categorical type of location where the homicide occurred, and whether the homicide was gang-related (yes = 1, no = 0). Circumstances preceding the homicide included whether the homicide was precipitated by another crime (yes = 1, no = 0) and the categorical nature of the other crime.

### 2.3 Analysis plan

We calculated an aggregate homicide rate across the sample of 32 states and D.C. using 2017 5-year population estimates from the ACS, with separate rates calculated by foreign-born status and nation of birth. Homicide rates were calculated by dividing the number of homicide victims in each state by the residential population and multiplying by 100,000. Next, chi-squared and analysis of variance (ANOVA) tests were used to compare foreign-born and U.S.-born homicide victims by victim and incident characteristics. Finally, we estimated a multilevel mixed-effects logistic regression model predicting whether a decedent is foreign-born, nesting 8,593 persons in 32 states and D.C.^6^. This allowed for examination of the unique variance of each measure by foreign-born status. Analyses were conducted in STATA 16.

## 3 Results

### 3.1 Prevalence of foreign-born homicide victims

The homicide rate for foreign-born victims, displayed in Table 2, was 3.28 per 100,000 population, compared to 5.60 for U.S.-born victims. Overall, individuals born in other countries experienced a lower homicide rate than individuals born in the U.S. In terms of nation of birth, victims born in Honduras had the highest homicide rate at 11.00 per 100,000 residents, followed by those born in El Salvador at 5.98 and Jamaica at 5.70.

**Table 2:** 2017 homicide rates by foreign-born status and nation of birth per 100,000 population using the NVDRS and U.S. Census (N = 9,428).

		# homicide victims	2017 sample population	Rate per 100,000
Foreign-born status	Foreign-born	730	22,227,714	3.28
	U.S.-born	8,698	154,766,348	5.60
				
Nation of birth	Honduras	33	300,059	11.00
	El Salvador	51	852,279	5.98
	Jamaica	26	456,496	5.70
	Guatemala	29	576,648	5.03
	Mexico	207	4,527,636	4.57
	Dominican Republic	36	855,409	4.21
	Vietnam	12	515,113	2.33
	China	21	1,518,956	1.38
	India	17	1,259,703	1.35
	Other countries	268	12,902,238	2.08
	Missing/Unknown	30		

### 3.2 Victim characteristics

Table 3 presents differences between U.S. and foreign-born victims across victim characteristics. Age was statistically significant, with foreign-born victims being older than U.S.-born victims. Although the majority of homicide victims were male in both groups, almost one quarter of foreign-born victims were female, compared to 21% of U.S.-born victims; this difference was statistically significant. There was also a significant difference with respect to marital status. About 34% of foreign-born victims were married, compared to only 15% of U.S.-born victims. U.S.-born victims were more likely to be White (31% versus 13%) or Black (54% versus 14%) and less likely to be Hispanic (11% versus 56%) or Asian (0.6% versus 14%). U.S.-born and foreign-born victims also significantly differed in their educational attainment. We found that 33% of U.S.-born victims had less than a high school degree, compared to 39% of foreign-born victims. In addition, more foreign-born victims had a bachelor's degree or higher (11%), compared to U.S.-born victims (5%). U.S.-born victims were significantly more likely to have a mental health diagnosis (5% versus 2%) when compared to foreign-born victims.

**Table 3:** Victim characteristics by foreign-born status using a 2017 NVDRS sample (N = 9,428).

		U.S.-born		Foreign-born		Difference
		n	%	n	%	
Age	0–14	454	5.22	5	0.68	**
	15–24	2,167	24.91	121	16.58	
	25–34	2,495	28.68	197	26.99	
	35–54	2,482	28.54	275	37.67	
	55 and up	1,100	12.65	132	18.08	
Sex	Male	6,879	79.09	552	75.62	*
	Female	1,819	20.91	178	24.38	
Marital status	Married	1,276	14.67	245	33.56	**
	Not married	7,319	84.15	473	64.79	
	Unknown	103	1.18	12	1.64	
Race/Ethnicity	White	2,708	31.13	96	13.15	**
	Black	4,701	54.05	108	14.79	
	Asian/Pacific Islander	52	0.60	104	14.25	
	Other/unspecified	284	3.27	14	1.92	
	Hispanic	952	10.95	406	55.62	
	Unknown	1	0.01	2	0.27	
Education	Below high school	2,823	32.46	284	38.90	**
	High school degree/GED	3,927	45.15	251	34.38	
	Some college credit	1,002	11.52	59	8.08	
	Associate degree	365	4.20	33	4.52	
	Bachelor degree/higher	430	4.94	81	11.10	
	Unknown	151	1.74	22	3.01	
Mental health diagnosis	Yes	427	4.91	17	2.33	**
	No	8,271	95.09	713	97.67	
Alcohol problem	Yes	311	3.58	22	3.01	–
	No	8,387	96.42	708	96.99	
Substance abuse problem	Yes	1,046	12.03	71	9.73	–
	No	7,652	87.97	659	90.27	
Total		8,698	92.26	730	7.74	

p < .01 = ** p < .05 = * n.s. = -

### 3.3 Incident and circumstance characteristics

Table 4 shows our findings related to incident and circumstance characteristics. There were significant differences in the types of locations at which U.S.-born and foreign-born victims were injured. While U.S.-born victims were more likely to be injured in a house or apartment (47% versus 40%), foreign-born victims were more likely to be injured on a street or sidewalk or in an alley (23% versus 20%), in a public use area (12% versus 8%), or in other areas (22% versus 19%). Additionally, U.S.-born victims (6%) were significantly more likely to have used a weapon during the incident in which they were killed than foreign-born victims (4%).

**Table 4:** Incident and circumstance characteristics by foreign-born status using a 2017 NVDRS sample (N = 9,428).

		U.S.-born		Foreign-born		Difference
		n	%	n	%	
Victim–suspect relationship	Current or former partner	791	9.09	97	13.29	–
	Family member	743	8.54	37	5.07	
	Acquaintance	1,818	20.90	132	18.08	
	Stranger	516	5.93	81	11.10	
	Other	36	0.41	1	0.14	
	Unknown	4,794	55.12	382	52.33	
Method/weapon	Firearm	6,344	72.94	419	57.40	–
	Sharp instrument	888	10.21	154	21.10	
	Blunt object	412	4.74	39	5.34	
	Hanging, strangulation	220	2.53	29	3.97	
	Other	640	7.36	74	10.14	
	Unknown	194	2.23	15	2.05	
Location of injury	House, apartment	4,123	47.40	289	39.59	**
	Street, sidewalk, alley	1,777	20.43	164	22.47	
	Public use area	666	7.66	86	11.78	
	Other	1,680	19.31	157	21.51	
	Unknown	452	5.20	34	4.66	
Victim used a weapon	Yes	476	5.47	27	3.70	*
	No	8,222	94.53	703	96.30	
Gang-related	Yes	668	7.68	65	8.90	–
	No	8,030	92.32	665	91.10	
Homicide precipitated by another crime	Yes	2,141	24.61	185	25.34	–
	No	6,557	75.39	545	74.66	
Nature of the othercrime (most serious)	Drug trade	271	12.66	12	6.49	–
	Robbery	528	24.66	84	45.41	
	Burglary	243	11.35	17	9.19	
	Motor vehicle theft	54	2.52	3	1.62	
	Arson	24	1.12	0	0.00	
	Rape, sexual assault	46	2.15	5	2.70	
	Gambling	7	0.33	0	0.00	
	Assault, homicide	795	37.13	49	26.49	
	Witness tampering	8	0.37	3	1.62	
	Other	131	6.12	6	3.24	
	Unknown	34	1.59	6	3.24	

p < .01 = ** p < .05 = * n.s. = -

Nature of the other crime (most serious) accounts for all homicides precipitated by another crime (n = 2,326)

Our analysis was limited by the number of unknown relationships between suspects and victims (55% of cases involving U.S.-born victims and 52% involving foreign-born victims). With this limitation in mind, foreign-born victims were more likely to be killed by a current or former intimate partner (13% versus 9%) or a stranger (11% versus 5%) when compared to U.S.-born victims. Conversely, U.S.-born victims were more likely to be killed by a family member (8% versus 5%) or an acquaintance (21% versus 18%). These differences, however, were not statistically significant. Similarly, while not statistically significant, about 73% of U.S.-born victims were killed with a firearm, compared to only 57% of foreign-born victims. Further, while there was no significant difference in victims' foreign-born status with respect to gang-related homicides, there were few gang-related homicides involving foreign-born victims (n = 65).

### 3.4 Multilevel logistic regression results

Finally, Table 5 presents unstandardized coefficients and odds ratios from a multilevel mixed-effects logistic regression model predicting foreign-born status among homicide victims, nested within states. Approximately 6% of the variance in foreign-born homicide victimization occurred at the state level. Regarding victim characteristics, foreign-born decedents were more likely to be older, married, and Asian/Pacific Islander or Hispanic. Examining educational attainment, foreign-born decedents were less likely to have a high school degree/GED or some college credit, compared to less than a high school degree. Finally, in comparison to U.S.-born homicide victims, foreign-born victims were less likely to have a mental health diagnosis or substance abuse problems.

**Table 5:** Multilevel mixed-effects logistic regression predicting foreign-born homicide victimization using a 2017 NVDRS sample (N = 8,597).

		b (se)	OR
**Victim characteristics**			
Age	0–14	ref	ref
	15–24	1.59 (0.51)**	4.91
	25–34	2.02 (0.51)**	7.49
	35–54	2.26 (0.51)**	9.61
	55 and up	2.63 (0.51)**	13.82
Sex (male)		−0.06 (0.14)	0.94
Married		0.84 (0.12)**	2.30
Race/Ethnicity	White	ref	ref
	Black	−0.20 (0.17)	0.82
	Asian/Pacific Islander	4.04 (0.24)**	57.02
	Other/unspecified	0.62 (0.33)	1.86
	Hispanic	2.84 (0.15)**	17.16
Education	Below high school	ref	ref
	High school degree/GED	−0.42 (0.11)**	0.66
	Some college credit	−0.54 (0.18)**	0.58
	Associate degree	−0.36 (0.25)	0.70
	Bachelor degree/higher	0.34 (0.20)	1.40
Mental health diagnosis		−0.62 (0.30)*	0.54
Alcohol problem		−0.17 (0.29)	0.85
Substance abuse problem		−0.38 (0.17)*	0.68
		
**Incident/circumstance characteristics**			
Victim–suspect relationship	Current or former partner	ref	ref
	Family member	−0.42 (0.25)	0.66
	Acquaintance	−0.15 (0.21)	0.86
	Stranger	0.22 (0.24)	1.24
	Other	−1.23 (1.11)	0.29
	Unknown	0.02 (0.20)	1.02
Method/weapon	Firearm	ref	ref
	Sharp instrument	0.69 (0.14)**	2.00
	Blunt object	0.05 (0.24)	1.05
	Hanging, strangulation	0.75 (0.27)**	2.11
	Other	0.30 (0.19)	1.35
Location of injury	House, apartment	ref	ref
	Street, sidewalk, alley	0.18 (0.14)	1.19
	Public use area	0.52 (0.17)**	1.68
	Other	0.29 (0.14)*	1.33
Victim used a weapon		−0.40 (0.25)	0.67
Gang-related		−0.55 (0.18)**	0.58
Homicide precipitated by another crime		0.15 (0.13)	1.16

Groups = 33 Wald chi = 918.14** State variance = 6.39% R-squared from single-level model = 0.32.

p < .01 = ** p < .05 = * ref = reference category.

Missing: Married (n = 115), Race (n = 3), Education (n = 173), Method/weapon (n = 209), Location of injury (n = 486).

Similar to our bivariate comparisons, limited significant associations with foreign-born status emerged among incident and circumstance characteristics. Foreign-born homicide victims were more likely to be killed by a family member, compared to a current or former intimate partner. Similarly, they were significantly more likely to be killed with a sharp object or by hanging or strangulation as opposed to a firearm. Compared to being killed in a house or apartment, foreign-born victims were significantly more likely to be killed in a public use area. Finally, we found lower odds of foreign-born homicide victims being killed in gang-related incidents compared to U.S.-born victims.

## 4 Discussion

The present study sought to examine the scope and nature of foreign-born homicide victimization in the U.S. This study is one of the first to broadly examine these issues using the NVDRS, arguably the most comprehensive source of homicide data in the U.S. It relied on data collected in 2017 encompassing more than 9,000 homicide victims in 32 states and D.C. Given prior research suggesting that the foreign-born population is hesitant to report victimization, our examination of homicide victimization offers an alternative method of estimating violent victimization among this population that can be used to inform prevention efforts.

The findings revealed that 8% of homicide victims in the sample were foreign-born. Further, the homicide victimization rate of foreign-born individuals (3.28 per 100,000) was lower than that of U.S.-born individuals (5.60 per 100,000). While foreign-born persons are at reduced risk of homicide victimization overall, foreign-born individuals from some nations are at substantially higher risk of victimization. We found that those born in Honduras (11.00 per 100,000), El Salvador (5.98 per 100,000), and Jamaica (5.70 per 100,000) experienced higher homicide rates than those born in the U.S. Residents in these same nations experience some of the world’s highest homicide victimization rates (The World Bank, 2018).

Victimization rates among those born in Honduras were substantially higher than those born in other countries, and these rates were nearly twice as high as U.S.-born residents. A high number of Hondurans immigrated to the U.S. during the decade prior to the study period. From 2007 to 2015 alone, there was a 32% increase in foreign-born residents from Honduras (Cohn et al., 2017). Many of these Honduran immigrants fled to the U.S. because of the high levels of violence, poverty, unemployment, and problems associated with governance afflicting Honduras (Landa-Blanco et al., 2020, Médecins Sans Frontiers, 2019). The high rates of homicide victimization among Honduran-born victims may be related to several factors that deserve further inquiry, such as the crime-prone demographic profile of these immigrants (e.g., younger, male), cultural conflict, and economic deprivation. Further analysis is needed to determine the factors associated with the variation between homicides involving foreign-born victims from Honduras and those involving foreign-born victims from other nations.

The homicide victimization rates of individuals from El Salvador and Jamaica were also higher than the rates of U.S.-born victims and victims from most other nations. Like Honduras, El Salvador is located in the Northern Triangle region of Central America and is consistently ranked as one of the most violent places in the world (The World Bank, 2018). Youth participating in Youth Outreach Centers across at-risk neighborhoods in El Salvador indicated that the majority felt unsafe where they lived; 61% reported a homicide occurring in their neighborhood in the previous year. Almost two-fifths of this sample indicated that they planned to migrate in the next three years, and youth engaging in risky behaviors were more likely to plan to migrate (Roth and Hartnett, 2018). Nearby in the Caribbean, Jamaica likewise commonly experiences one of the highest homicide rates in the region and world. In recent years, research has attributed much of this violence to problems associated with political corruption, poor education systems, unemployment, and concentrated disadvantage (The World Bank, 2018, Katz and Maguire, 2015). While the current dataset limits our investigation of these issues, the ties between migration and prior community characteristics, in particular exposure to violence and other traumatic events, warrants further research.

Theorists have long speculated on cultural influences on offending. In support of these theories, cross-national research reinforces hypotheses of a cultural component influencing violence in Latin America (Nivette, 2011, Cao and Zhang, 2017). Scholars have speculated that the high violence rates are impacted by the culture of “machismo” (e.g., strong, sometimes aggressive, masculinity; Neapolitan, 1994) or societal organization around alcohol and street gangs (Cole and Gramajo, 2009). Further, countries in the region possess legacies of colonialism, slavery, and poor governance (Cao and Zhang, 2017). This occurs in a context in which outside nations, notably the U.S., have played a contributing role in destabilizing the region, for example, through the war on drugs (Youngers and Rosin, 2005). It is possible that these native cultural influences may shape the situations to which victims are exposed and their risk of victimization in the U.S.

In terms of victim characteristics, demographic shifts of the U.S. foreign-born population may be contributing to the overall lower homicide victimization rate among foreign-born persons. Recently arrived immigrants are more likely to be Asian, educated, and female (Radford, 2019)—demographic groups that our study and others find are associated with lower rates of violence (Fowler et al., 2018). Consistent with these findings, we found that foreign-born homicide victims were more likely to be Hispanic or Asian, female, older, married, and either have a college degree or lack a high school diploma or GED. In addition, foreign-born victims were less likely to have been diagnosed with a mental health problem, compared to U.S.-born victims. In contrast to victim characteristics, we identified few significant differences for incident or circumstance characteristics.

Finally, foreign-born victims were less likely to be killed in a gang-related incident than U.S.-born victims. In fact, foreign-born persons were rarely the victims of gang-related homicide. In 2017, there were only 65 foreign-born gang-related homicide victims (0.7%) among our sample of 9,428 homicide victims. In contrast, over 650 gang-related homicide victims were U.S.-born (7% of the total sample). This finding suggests that foreign gangs and foreign-born gang members may play a small role in the nation's overall homicide problem. This contrast also extends to drug-related crimes. Only 7% of foreign-born homicide victims were killed in incidents preceded by drug trade, compared to 13% of U.S.-born victims. The growth of gangs such as Mara Salvatrucha (MS-13) with strong international ties has served to connect immigration issues with criminality in the minds of some of the public. Despite this, our findings do not support assertions that foreign-born homicides are largely driven by gang- and drug-related violence. While our study only examined homicide victimization and cannot speak to foreign-born homicide offending, past research has found that most homicides are intra-racial, with victims and offenders sharing the same racial/ethnic backgrounds (Hewitt, 1988). Thus, future research examining homicide offending among the foreign-born could identify similar trends.

Despite the strengths of our dataset, limitations exist. First, the methodology relies on official data collected within each state; this, in turn, relies heavily upon self-reports by the victim's associates and family to a law enforcement officer or death investigator, which could lead to underreporting or inaccuracies. Second, the inclusion of the additional states in our analysis, potentially with distinct foreign-born population compositions, could alter our results. Further, our results should be interpreted with caution on measures with high amounts of missing data (e.g., the victim–suspect relationship). Third, we allow for variation at the state level in our analysis given differing migration patterns, environments, and policies. Future work should examine whether alternative levels of geography (e.g., counties or cities) better inform foreign-born homicide victimization. Last, our measure of gang-involved homicides is limited by variation in law enforcement's definitions of gang involvement and overreliance on official law enforcement reports (Frazier et al., 2017).

With the number of immigrants in the country growing each year, having reliable estimates of violent victimization among this population as well as risk and protective factors influencing this victimization remains relevant to ensure that violent crime prevention resources are data-driven and utilized efficiently. The present study indicates significant differences between foreign-born and U.S.-born homicide victims which serve as starting points for further inquiry and prevention efforts.

## Author contributions

KF and MG conducted the statistical analyses and assisted in drafting the manuscript. JH, HC, DC, and TC assisted in drafting and critically reviewing and revising the manuscript. CK conceptualized the idea for the study, acquired the data from the CDC, coordinated the work conducted by the authors, developed the analysis plan, participated in interpretation of the analyses, and assisted drafting and revising the manuscript.

## Declaration of Competing Interest

The authors declare that they have no known competing financial interests or personal relationships that could have appeared to influence the work reported in this paper.

## Footnotes

1 The number of states participating in NVDRS has increased significantly by year over the past decade. We used 2017 data because it was the most recent data available, included the greatest number of states, and eliminated issues related to a changing sampling frame.

2 Non-participating states included Alabama, Arkansas, Florida, Hawaii, Idaho, Louisiana, Mississippi, Missouri, Montana, Nebraska, North Dakota, South Dakota, Tennessee, Texas, and Wyoming. Approximately 34% of foreign-born individuals in the U.S reside in the states not included in our sample. Immigrants in the sample states comprise an average of 2% of their total population compared to an average of 1.9% non-participating states (Migration Policy Institute, 2019).

3 In calculating the national homicide rate and in the state-level multilevel analysis, we rely on the population totals from these four counties rather than the statewide population because the California NVDRS program restricts data collection to these counties.

4 5-year estimates were used given the smaller margins of error.

5 We relied on pre-coded variables; narratives were not reviewed for missing independent variables.

6 Four states reported fewer than 20 homicides in 2017. We reran our analysis with these cases removed and found no substantive changes in the results; as such, results including all available states are reported. Correlation coefficients, the variance inflation factor (VIF; 1.13), and conditional index scores (14.82) did not identify the presence of multicollinearity in our final model.

## References

[b0005] Armenta A., Rosales R. (2019). Beyond the fear of deportation: understanding unauthorized immigrants’ ambivalence toward the police. Am. Behav. Scientist.

[b0010] Budiman, A. (2020, August 20). Key findings about U.S. immigrants. Pew Research Center. Retrieved from https://www.pewresearch.org/fact-tank/2020/08/20/key-findings-about-u-s-immigrants/.

[b0015] Cao L., Zhang Y. (2017). Governance and regional variation of homicide rates: evidence from cross-national data. Int. J. Offender Therapy Comparative Criminol..

[b0020] Centers for Disease Control and Prevention, 2018. National Violent Death Reporting System (NVDRS) Coding Manual Revised [Online] 2018 National Center for Injury Prevention and Control, Centers for Disease Control and Prevention (producer). Retrieved from https://www.cdc.gov/injury.

[b0025] Cepeda A., Negi N., Nowotny K., Arango J., Kaplan C., Valdez A., Kubrin C.E., Zatz M.S., Martinez R. (2012). Punishing Immigrants: Policy, Politics, and Injustice.

[b0030] Cohn, D., Passell, J., Gonzalez-Barrera, A., 2017. Rise in U.S. immigrants from El Salvador, Guatemala and Honduras outpaces growth from elsewhere. Pew Research Center. Retrieved from https://www.pewresearch.org/hispanic/2017/12/07/rise-in-u-s-immigrants-from-el-salvador-guatemala-and-honduras-outpaces-growth-from-elsewhere.

[b0035] Cole J.H., Gramajo A.M. (2009). Homicide rates in a cross-section of countries: Evidence and interpretations. Population and Development Review.

[b0040] Dews, F. (2018, July 19). What percentage of U.S. population is foreign born? Retrieved from https://www.brookings.edu/blog/brookings-now/2013/10/03/what-percentage-of-u-s-population-is-foreign-born/.

[b0045] Eggers A.S., Mitchell O. (2016). The emotional guardianship of foreign-born and native-born Hispanic youth and its effect on violent victimization. Race and Justice.

[b0050] Fowler, K. A., Jack, S. P. D., Lyons, B. H., Betz, C. J., & Petrosky, E. (2018). MMWR Surveillance Summaries – 2018. Retrieved from https://www.ncbi.nlm.nih.gov/. 10.15585/mmwr.ss6702a1 PMC5829936 29389917

[b0055] Frazier L., Ortega L., Patel N., Barnes J., Crosby A.E., Hempstead K. (2017). Methods and findings from the National Violent Death Reporting System for identifying gang-like homicides, 2005–2008. J. Natl Med. Assoc..

[b0060] Grace B.L., Bais R., Roth B.J. (2018). The violence of uncertainty — Undermining immigrant and refugee health. N. Engl. J. Med..

[b0065] Hewitt J.D. (1988). The victim-offender relationship in convicted homicide cases: 1960–1984. J. Criminal Justice.

[b0070] Jones, J. M. (2019, June 21). New high in U.S. say immigration most important problem. Gallup. Retrieved from https://news.gallup.com/poll/259103/new-high-say-immigration-important-problem.aspx.

[b0075] Katz C.M., Maguire E.R., Harriott A., Katz C.M. (2015). Gangs in the Caribbean: Responses of State and Society.

[b0080] Landa-Blanco M., Cheon H., Reyes Flores L.G., Spohn C., Katz C.M. (2020). Violence in Honduras from 2008 to 2018. Injury Prevention.

[b0085] Médecins Sans Frontiers. (2019). Forced to flee Central America's Northern Triangle: A neglected humanitarian crisis, 2017. Retrieved from http://urbanspaces.msf.org/wp-content/uploads/2019/03/forced-to-flee-central-americas-northern-triangle_-a-neglected-humanitarian-crisis.pdf.

[b0090] Migration Policy Institute (MPI) tabulation of data from U.S. Census Bureau, 2010-2017 American Community Surveys (ACS), and 1970, 1990, and 2000 Decennial Census. All other data are from Campbell J. Gibson and Emily Lennon, “Historical census statistics on the foreign-born population of the United States: 1850 to 1990” (Working Paper no. 29., U.S. Census Bureau, Washington, D.C., 1999).

[b0095] Migration Policy Institute (MPI). (2019). U.S. immigrant population by state and county. Retrieved from https://www.migrationpolicy.org/programs/data-hub/charts/us-immigrant-population-state-and-county.

[b0100] Neapolitan J.L. (1994). Cross-national variation in homicides: the case of Latin America. Int. Crim. Justice Rev..

[b0105] Nivette A.E. (2011). Cross-national predictors of crime: a meta-analysis. Homicide Studies.

[b0110] Radford J. (2019).

[b0115] Roth B.J., Hartnett C.S. (2018). Creating reasons to stay? Unaccompanied youth migration, community-based programs, and the power of “push” factors in El Salvador. Children and Youth Services Review.

[b0120] Singh G.K., Hiatt R.A. (2006). Trends and disparities in socioeconomic and behavioural characteristics, life expectancy, and cause-specific mortality of native-born and foreign-born populations in the United States, 1979–2003. Int. J. Epidemiol..

[b0125] Sommers I., Fagan J., Baskin D. (1994). The influence of acculturation and familism on Puerto Rican delinquency. Justice Q..

[b0130] Sorenson S.B., Shen H. (1996). Homicide risk among immigrants in California, 1970 through 1992. Am. J. Public Health.

[b0135] The World Bank. (2018). Intentional homicide rates. Retrieved from https://data.worldbank.org/indicator/VC.IHR.PSRC.P5?most_recent_value_desc=true.

[b0140] U.S. Census Bureau. (2019). About foreign born. Retrieved from https://www.census.gov/topics/population/foreign-born/about.html#par_textimage.

[b0145] Youngers C.A., Rosin E. (2005).

